# Swallowing function in patients with spinal muscular atrophy before and after the introduction of new gene-based therapies: what has changed?

**DOI:** 10.1007/s10072-024-07883-0

**Published:** 2024-12-04

**Authors:** Marta Ruggiero, Gabriele Giannotta, Greta Pirani, Federica Saponaro, Maria Carmela Oliva, Camilla Ferrante, Antonio Trabacca

**Affiliations:** 1 Unit for Severe Disabilities in Developmental Age and Young Adults (Developmental Neurology and Neurorehabilitation), Associazione “La Nostra Famiglia” – IRCCS “E. Medea” - Scientific Hospital for Neurorehabilitation, Brindisi, Italy; 2 Scientific Institute I.R.C.C.S. “E. Medea”, Scientific Direction, Via Don L. Monza 20, Bosisio Parini (LC), 23842 Italy

**Keywords:** Spinal muscular atrophy, Swallowing, Swallowing assessment, Dysphagia, Scoping review

## Abstract

**objective:**

Individuals diagnosed with Spinal Muscular Atrophy (SMA), particularly those presenting with the most severe phenotypes, have long contended with significant swallowing dysfunction. The recent emergence of efficacious advanced therapy has fundamentally altered the landscape of SMA management. By encompassing both the pre and post gene-based therapy eras within our analysis, we endeavour to elucidate the potential impact of these novel therapeutic interventions on this function.

**Methods:**

Following the established methodology outlined by the Joanna Briggs Institute, a scoping review was conducted. This review encompassed relevant literature published up to March 2024. Two electronic databases were searched, with additional studies identified by reviewing reference lists of pertinent articles. The search strategy employed a combination of keywords including “spinal muscular atrophy”, “SMA”, “swallowing”, “feeding”, and “nutrition”. Articles were initially screened based on title and abstract, followed by a full-text review of eligible studies published in peer-reviewed English language journals.

**Results:**

The initial database search resulted in 462 articles, from which 23 studies were ultimately selected for analysis. Pre gene-based therapy studies revealed swallowing dysfunction as a prominent feature. Patients frequently reported challenges with chewing, choking on solids and liquids, and abnormal tongue movements during eating. Early dysphagia research relied on subjective measures (questionnaires, scales). objective measures like video fluoroscopic (VFSS) were rare. After gene-based therapies (GBTs), VFSS became the dominant, more objective method. Studies investigating the post-gene therapy era suggest potential clinical benefits, with trends towards improvement or stabilization of swallowing function.

**Conclusion:**

Pre gene-based therapy studies revealed widespread swallowing dysfunction in SMA. Conversely, the post-treatment era suggests potential improvement. Future research should prioritize identifying optimal therapies for individual swallowing function and develop validated assessments to optimize SMA management.

## Introduction

Neuromuscular diseases (NMDs) are a heterogeneous group of disorders, mostly with a genetic origin, primarily characterized by muscle weakness in one or more muscle groups. This weakness can lead to various complications, including contractures, scoliosis, problems with walking and hand function, difficulty swallowing (dysphagia), slurred speech (dysarthria), malnutrition, and respiratory failure [1, 2]. Spinal Muscular Atrophy (SMA) is a rare, autosomal recessive neuromuscular disease caused by deletions or mutations in the survival motor neuron (SMN1) gene leading to degeneration of alpha-motor neurons in the spinal cord with an estimated prevalence of 2.12/100,000 [3]. This degeneration leads to slow, progressive muscle weakness and wasting (atrophy) of skeletal muscles [4]. Historically, SMA has been classified into five major phenotypes (four affecting children) that are separated by the age at symptom onset and maximal motor milestone achieved [5].

Advances in therapeutic options, clinical research, trials, and real-world evidence have hastened progress in understanding the natural history of SMA, prompting a revision of the SMA phenotypes. Presently, SMA phenotype is viewed as a continuum, emphasizing the current functional status and response to therapy. Thanks to gene-based therapies (GBTs), it is now possible for a patient diagnosed with SMA type I to attain the functional capabilities of someone with SMA type II, III, or IV. Similarly, a patient with SMA type II can now achieve the functional abilities typically associated with SMA type III or IV.

Different historical subtypes span from type 0, emerging before birth (in utero), to type 4, which emerges in adulthood. Among these, the most prevalent are SMA types 1, 2, and 3 [6]. SMA type 1 typically begins between birth and six months, characterized by patients who are never able to sit without supports. This type can be further classified into subtypes 1a (symptoms present at birth), 1b (symptoms begin before 3 months), and 1c (onset between 3 and 6 months) [7]. Types 2 and 3 SMA are more heterogeneous and less severe than type 1 [8]. This population type is able to sit or to stand and walk independently, although this abilities may be lost later in the progression of the disease [9, 10].

Weakness in both the bulbar muscles (involved in swallowing) and the gastrointestinal tract muscles in SMA patients was documented [11, 12]. This dysfunction can contribute to feeding difficulties, including swallowing problems with aspiration risk, chewing difficulties, and weight issues (both underweight and overweight) [13–17]. Feeding disorders encompass difficulties with various aspects of food intake, including sucking during infancy, utensil use, mastication (chewing), and independent cup drinking [18]. Swallowing disorders, also known as dysphagia, are characterized by impairments within one or more of the four distinct stages of the normal swallowing process: the oral preparatory phase, oral transport phase, pharyngeal phase, and esophageal phase [19].

These feeding difficulties are most frequent in SMA type 1, less common in type 2 [11, 14, 17, 20, 21], and rarely seen in type 3 [14, 20, 21].

With advancements in care standards [5, 6] and the introduction of novel therapeutic approaches, these patients have seen powerful improvements in survival and functional abilities, altering the disease’s course [7–10]. However, most research has focused on survival and motor/respiratory outcomes [11]. Despite feeding being crucial for SMA patients, changes in their swallowing abilities haven’t received as much attention [22–28].

This literature review aims to provide an overview of the known swallowing issues in various types of SMA patients and to describe the changes observed from the pre-GBTs era to the current era of GBTs.

## Methods

### objective

The primary goal of this scoping review was to comprehensively evaluate and compare the swallowing function clinical changes observed in Spinal Muscular Atrophy (SMA) patients before and after the introduction of GBTs. This objective was driven by the transformative impact of GBTs on the treatment of SMA, which had heightened interest in understanding its clinical effects. To address the exploratory nature of this research question, the authors employed a scoping review methodology. This approach aligns with the Joanna Briggs Institute (JBI) methodology as detailed in their online reviewer’s manual for scoping reviews. The review adhered to the Preferred Reporting Items for Systematic Reviews and Meta-Analyses extension for Scoping Reviews (PRISMA-ScR) checklist to ensure transparency and completeness. While a pre-registered a priori protocol was not utilized, as it is not mandatory for scoping reviews. Further details regarding the review process can be obtained by contacting the corresponding author upon request.

### Inclusion and exclusion criteria

Inclusion criteria for articles selection in this review were: studies investigating any subtype of SMA (types I, II, III, or IV) in treated or untreated patients with varying degrees of severity, employing quantitative or qualitative assessments of swallowing abilities; and articles published in English. Exclusion criteria encompassed: studies focused on body mass or caloric intake; review articles; case reports; conference posters; and editorials.

### Search strategy

To achieve this objective, a comprehensive search strategy was employed to identify relevant literature. The search string was carefully crafted to ensure the inclusion of pertinent articles while minimizing irrelevant results. A comprehensive search was carried out, encompassing studies from PUBMED/Medline and Embase. Search terms included ‘spinal muscular atrophy’ AND (‘swallow’ oR ‘feeding’ oR ‘nutrition’). To ensure a comprehensive review of recent findings, we restricted our search to articles published between 2004 and 2024. This timeframe captured the latest advancements in SMA treatment options while maintaining a manageable selection of studies for analysis. Research published before 2004 was likely to be outdated or lack relevance to current treatment approaches.

### Study screening and selection

An initial screening of articles based on titles and abstracts was conducted according to the pre-defined criteria (see Inclusion/Exclusion Criteria section). Two reviewers performed this process independently. Duplicates were removed, and only full-text articles published in peer-reviewed English-language journals were considered. A third independent reviewer resolved disagreements arising during this double-blind review process using the Rayyan QCRI web application.

### Data synthesis

Following individual analysis of data extracted from each article subgroup, a qualitative synthesis was employed to compare swallowing function outcomes reported in the pre- and post-gene therapy subgroups. This comparative analysis aimed to elucidate the impact of GBTs on the clinical course of SMA.

## Results

The initial database research yielded 462 articles. Following the elimination of duplicates (*n* = 78) and the initial screening based on titles and abstracts, 363 records were excluded. After screening and selecting studies, a total of 21 articles were incorporated into the review. Among the studies included, based on the eligibility criteria, additional studies (*n* = 2) not identified in the initial search but found through cross-referencing some of the included articles were further added. Following the application of our inclusion criteria, the final number of studies selected for analysis was 23. The articles’ general information, participants, evaluation protocol, and main findings are presented in Table 1. The retrieved articles were then meticulously screened and categorized into two distinct subgroups based on the abstract information: pre-GBTs and post-GBTs. This categorization allowed for a targeted analysis of the clinical changes observed in each era. Table 2 presents a comparison of the two study groups.

**Table 1:** Articles’ general information, participants, evaluation protocol, and main findings

Untreated patients				
Title, author, year	Partecipants (*N*) inclusion/exclusion criteria	Population	outcome measures, units, time of follow-up	Main *r* esults
Feeding problems and malnutrition in spinal muscular atrophy type II Messina S. et al. [35]	Genetically and clinically confirmed diagnosis of spinal muscular atrophy (SMA) type 2.	122 SMA 2 patients 6 Groups: 1,1/47,0 years	*- Questionnaire on feeding difficulties* The questionnaire was designed by a consensus of neurologists, paediatricians, and speech therapists. Feeding abilities, including duration to complete a meal and feeding difficulties.	Chewing problems in 34/122 patients; more present in older than 20 years old; − 7 patients (6%) chocking episodes with both solid and liquid; − 14 chocking solid (11%) − 2 chocking liquid (2%) 7 patient complained about solid difficulties (6%) Swallowing difficulties affected 25% of patients, more frequently those over 20, often involving recurrent choking on solids or both solids and liquids.
Dysphagia in spinal muscular atrophy type ii more than a bulbar problem? van den Engel-Hoek L. et al. [19]	Genetically and clinically confirmed diagnosis of SMA type 2.	6 SMA 2 children (all sitters). 6 healthy children.	- *Self compiled questionnaire on swallowing for different type of food*; - Swallowing was measured with *surface electromyography (sEMG) of the sonomyography (SMG)* and with a *video fluoroscopic swallow study (VFSS)*.	All patients showed: − abnormal tongue movements; − multiple swallow to clear the oral cavity; − lack of strength to swallow solid food; − Insufficient movement of the hyoid and movement of opening;
Adiposity is increased among high-functioning, non-ambulatory patients with spinal muscular atrophy Sproule D. M. et al. [21]	Genetically and clinically confirmed diagnosis of SMA type 2.	25 SMA type 2 children.	*- Parental Questionnaire adapted*: duration to complete a meal and feeding difficulties were recorded, categorized by phase: extrabuccal (opening mouth), anterior (keeping food in mouth, chewing), or posterior (choking, repeated swallowing attempts, food aspiration). - *Hammersmith Functional Motor Scale Expanded (HFMSE);*	of 25 subjects, 9 (36%) reported swallowing or feeding dysfunction (choking on liquids, semi-solids, or solids), including 7 of 18 (39%) low-functioning (HFMSE < 12) and 2 of 7 (29%) high-functioning (HFMSE ≥ 12) SMA type 2 subjects. one subject in each group had difficulty ‘opening mouth’. Dietary modification or supplementation was required in 10 of 18 (56%) low-functioning and 2 of 7 (29%) high-functioning subjects, totaling 12 of 25 (44%) of the cohort. Estimated meal times were similar between groups: 24.3 ± 11.2 min (low-functioning) vs. 27.9 ± 15.8 min (high-functioning).
Prevalence and risk factors for feeding and swallowing difficulties in spinal muscular atrophy types II and III Chen Y.S. et al. [42]	Exclusion criteria: SMA type 1 patients.	108 SMA patients: (60 type 2 SMA, 48 type 3 SMA);58 Male (M); 50 Female (F); 3–45 years Group 1: without any feeding or swallowing problems; Group 2: with feeding or swallowing problems.	*-Questionnaire for feeding and swallowing difficulties in spinal muscular atrophy*.	Nearly one-half of patients had at least one feeding of swallowing problems: - choking: 33 - chewing difficulties: 22 35 patients had prolonged mealtimes (+ 30 min). Type 2 SMA, both sitter and non-sitter status, respiratory management needs, and especially poor head control were significantly more common among those with feeding and swallowing difficulties than those without. These patients also had significantly lower body weight.
Bulbar muscle MRI changes in patients with SMA with reduced mouth opening and dysphagia Chen R. I. et al. [46]	Genetically and clinically confirmed diagnosis of SMA type 1 2 3 4.	145 Patients SMA Patients. 119 Health Control.	*- Magnetic Resonance Imaging (MRI) during the mouth opening* with the instruction to open it “as far as possible without pain”. [only on 12 patients vs. 1 Healthy control]; *- Questionnaire for swallowing difficulties*; *- HFMSE*; *- MRC* Strength Scale.	35% reports Dysphagia or choking; 14% in SMA 1–3 history of recurrent choking; Mouth opening reduced (≤ 35 mm) in: 57% of all patients: 100% SMA 1; 79% SMA 2; 50% SMA 3a; 7% SMA 3 b.
Bulbar problems self-reported by children and adults with spinal muscular atrophy van der Heul A.M.B. et al. [31]	Inclusion criteria: Genetically and clinically confirmed diagnosis of SMA type 1 2 3 4.	118 Patients.	*- Diagnostic list of dysphagia and dysarthria in Neuromuscular Disease (NMD);*	SMA 2 report jaw, mastication, swallowing and intelligibility problems; JAW problems equally reported in all types; Swallow was difficult in solid food, significant association between jaw and mastication problems; 55 patients took more than 30 min to finish the meal.
Trajectory of change in the swallowing status in spinal muscular atrophy type I Choi Y.A. et al. [29]	Inclusion criteria: Genetically and clinically confirmed diagnosis SMA TYPE 1 with age > 2 Exclusion criteria: Patients involved in investigational drug trials.	11 Patients Mean age 4 years 6 M 5 F	*- Neuromuscular disease status scale (NdSSS);* *- Video fluoroscopy*	Swallowing function generally deteriorated before 12 months of age, typically around six months—the median age when patients began tube feeding due to oral intake difficulties, recurrent pneumonia, or signs of aspiration during feeding. In the oral transport phase, poor bolus formation was observed in all but one SMA type I patient. In the pharyngeal phase, frequent impairments in laryngeal elevation and epiglottic closure increased the risk of aspiration.
Mastication in patients with spinal muscular atrophy types 2 and 3 is characterized by abnormal efficiency, reduced endurance, and fatigue van der Heul (A) M. (B) et al. [31]	Inclusion criteria: SMA types 2 e 3 who mentioned bulbar problems.	27 patient 18 SMA 2 9 SMA 3(13–67 years)	-*Diagnostic list of dysphagia and dysarthria;* *- The Functional oral Intake Scale (FoIS);* *- Test of mastication and swallowing solid;* *− 6 min mastication test;* *- Active mouth opening;* (maximal and unasstisted) *- Muscle ultrasound;*	Non-ambulant patients demonstrated inefficient mastication, indicated by median z-scores for masticatory cycles (z = 1.8), number of swallows (z = 4.3), and time to finish a cracker (z = 3.4). Limited endurance during continuous mastication was shown by the 6-minute mastication test (median z = − 1.5). Patients reported increased fatigue immediately after and 5 min post-test (*p* < 0.001; *p* = 0.003). Reduced maximal mouth opening was associated with mastication problems (*p* < 0.001). Muscle ultrasound revealed abnormal muscle structure in 90% of both ambulant and non-ambulant patients.
Swallowing problems in spinal muscular atrophy types 2 and 3: a clinical, videofluoroscopic and ultrasound study van der Heul A.M.B. et al. [31]	Inclusion criteria: SMA patients who mentioned mastication and/or swallowing problems; SMA patients who had not accessed to therapies.	27 Patients SMA 2 AND 3; 13 Years and older.	*- Diagnostic list of dysphagia and dysarthria;* *- FoIS;* *- Dysphagia limit;* *- Timed test swallowing;* *- Test of mastication and swallowing solid;* *- Video fluoroscopy;* *- Muscle ultrasound.*	The most common swallowing problem among non-ambulant patients was difficulty swallowing solid food. Two of three ambulant patients reported a sensation of food sticking in the throat. Non-ambulant patients exhibited piecemeal deglutition and pharyngeal residue after ingesting liquids. Additionally, eighteen non-ambulant patients showed increased echogenicity of the submental muscles and tongue, displaying a ‘moth-eaten’ pattern. An increased time to finish a standardized cracker was associated with a decreased Dysphagia Limit (DL).
**Treated patients**				
Health outcomes in spinal muscular atrophy type 1 following AVXS-101 gene replacement therapy Al-Zaidy S. et al. [25]	Inclusion criteria: Genetically and clinically confirmed diagnosis of SMA type 1.	12 Patients treated with onasemnogene.	- Swallowing function assessed with a *video fluoroscopic* swallow test. Follow-up on days 7, 14, 21, and 30 followed by monthly visits through 12 months post-dosing, and then every three months through two years post-dosing.	At baseline, 7 of 12 patients (58%) did not require feeding support, and by the end of the follow-up, 6 of these 7 continued without support. The proportion of patients able to safely swallow liquids increased from 4 of 12 (33%) at baseline to 10 of 12 (83%) at follow-up. overall, the number of patients capable of safe swallowing sufficient for at least partial oral feeding rose from 7 (58%) at baseline to 11 (92%) at the end of the follow-up period.
Feeding and swallowing problems in infants with spinal muscular atrophy type 1: an observational study van der Heul A.M.B. et al. [31]	Inclusion criteria: Genetically and clinically confirmed diagnosis of SMA type 1.	5 SMA type 1 patients undergoing Nusinersen treatment. 11 SMA patients with only supportive care	*- observation list* Item: ¬ *Fatigue related to oral feeding;* ¬ *Unsafe swallowing;* ¬ *Regurgitation of the food;* ¬ *Respiratory System.* *- Video fluoroscopy;* *- CHoP-INTEND* Wet breathing indicates unsafe swallowing.	NUSINERSEN GRoUP: onset of disease and initiation of tube feeding occurred at higher ages. After starting nusinersen, sucking and swallowing temporarily improved, but issues returned after 8–12 months. SUPPoRTIVE CARE GRoUP: Patients consistently failed to drink the recommended volume, exhibiting unsafe drinking and swallowing, with coughing and wet breathing during and after feeding. Treated and untreated patients showed similar score distributions in VFSS and CHoP-INTEND.
Evolution of bulbar function in spinal muscular atrophy type 1 treated with nusinersen Weststrate H. et al. [7]	Inclusion criteria: Genetically and clinically confirmed diagnosis of SMA. Patients undergoing Nusinersen treatment	24 Patients (14 females; 10 males) - SMA type 1a 3 - SMA type 1b 9 - SMA type 1c 12 Tube fed at baseline: - SMA type 1a 3/3 - SMA type 1b 5/9 - SMA type 1c 6/12	- *p-FoIS* at baseline, at 6 months after initiation of nusinersen, at 12 months, and at 24 months; -*Video fluoroscopy* for eight patients: 4 with type 1b SMA and 4 with type 1c. For all patients these were performed after starting nusinersen and at variable, unspecified time points; - CHoP-INTEND	*Video fluoroscopy*: 4 (1b) + 2 (1c) risk of aspiration and weak swallowing. Median p-FoIS for type 1a remained at 1 from baseline through all follow-up points. For type 1b, it declined from 3 at baseline to 1 at 6 months, then increased to 2 at 12 and 24 months. Type 1c patients had a median p-FoIS of 5 at baseline, which decreased to 2 at 6 months after starting nusinersen and rose to 3 at 12 and 24 months. Six patients showed a 1-point improvement in their p-FoIS scores: five improved from 1 to 2, and one type 1c patient improved from 2 to 3.
Assessment of bulbar function in adult Patients with 5q-SMA type 2 and 3 under treatment with nusinersen Brakemeier S. et al. [31]	Inclusion criteria: Patients with genetically and clinically confirmed SMA type 2 or 3 treated with Nusinersen. Bulbar dysfunction was documented before treat. initiation, and patients did not have a PEG.	22 SMA type 2 and 3 adult patients 13 M 9 F	-*Sydney Swallow Questionnaire (SSQ);* *- Amyotrophic Lateral Sclerosis Functional Rating Scale Revised (b-ALSFRSR);* *- HFMSE;* *- Revised Upper Limb Module (RULM)*.	Bulbar function measured by the ALS-FRS-R bulbar subscore showed no significant changes between T0 and T1 or T0 and T2. Similarly, SSQ scores did not significantly change over these periods. Although bulbar scores did not improve with nusinersen treatment, the absence of decline suggests a therapeutic effect on bulbar dysfunction.
oral and swallowing abilities tool (orSAT) in nusinersen treated patients Bert B. et al. [32]	Inclusion criteria: Genetically and clinically confirmed diagnosis of SMA patients treated with Nusinersen. (between 3 weeks and 15 months of age).	20 SMA1 infants 11 F 9 M	-*oral and Swallowing Abilities Tool (orSAT);* T0– before nusinersen start T1– after treatment ranged between 12 months and 62 months.	At treatment initiation, 12 of 20 infants had normal swallowing without the need for tube feeding. on follow-up, 10 of these 12 maintained normal swallowing, while the other 2 required tube feeding but regained partial oral intake. The remaining 8 infants already had tube feeding at baseline: 4 of them also had tracheostomy and showed no changes on the orSAT scale; the other 4, without tracheostomy, demonstrated partial functional improvement.
Flexible endoscopic evaluation of swallowing in children with type 1 spinal muscular atrophy Zang J. et al. [33]	Inclusion criteria: Genetically and clinically confirmed diagnosis of SMA type 1.	10 SMA patients 5 M 5 F 9/10 onasemnogene 1/10 Risdiplam 5/10 previously started with nusinersen	*- CHoP-INTEND;* *- orSAT;* *- Neuromuscular Disease Swallowing Status Scale (NdSSS);* *- Flexible endoscopic evaluation of swallowing (FEES)*; - *Penetration–Aspiration Scale (PAS);* Baseline + 6 Months	Common FEES findings included poor secretion management, delayed or absent swallowing reflex, spillage, penetration, aspiration, post-swallow residue, and reduced laryngeal sensitivity.Delayed swallowing or inability to swallow occurred in all children. At the first FEES, all but one child showed delayed initiation of the swallowing reflex; all children showed this delay at follow-up. At inclusion, four children had completely lost the ability to swallow; during follow-up, two of them began swallowing with significant delay. Weak sucking was observed in six cases at inclusion and five at follow-up, while sufficiently strong sucking was possible for one child at inclusion and two at follow-up.Feeding-related fatigue was seen in six of nine children at inclusion and in three of eight at the second examination.
Risdiplam in non-sitter patients aged 16 years and older with 5q spinal muscular atrophy Ñungo Garzon N. C. et al. [37]	Inclusion criteria: - Genetically and clinically confirmed diagnosis of SMA type 2 with Risdiplam in a single daily dose - All patients were ‘sitters’ but later lost this ability.	6 patients with type 2 SMA (> 16 years old);	*- RULM* *- Egen Klassifikation Scale version 2 (EK2);* *- ALFSR-R* *- Goal Attainment Scale (GAS);* *- Body Mass Index*. Baseline; 6 months; 12 months;	3 patients reported improved swallowing in functional scale scores, with two of whom showing clinically meaningful improvements in BMI. In one of this, percutaneous endoscopic gastrostomy (PEG) had been proposed due to sustained weight loss in previous visit but was no longer recommended after the improvement with Risdiplam. Improvements in the swallowing and eating domain scores of the EK2 and ALSFRS-R (but no effects in Rulm).
Longitudinal changes of swallowing safety and efficiency in infants with spinal muscular atrophy who received disease modifying therapies Leon-Astudillo C. et al. [49]	Inclusion criteria: Genetically and clinically confirmed diagnosis of SMA.	7 infants (0–12 months) (5 M; 2 F) - All subjects received nasemnogene; 3 received risdiplam in addition; 1 Nusinersen and Risdiplam in addition.	*- Video fluoroscopy;* *- Penetration–Aspiration* *Scale (PAS);* *- p-FoIS;* *- CHoP-INTEND;* *- Gross Motor outcome (GRo)*	PAS at diagnosis was abnormal in four subjects. Six subject required feeding modifications after VFSS results. of these three had silent aspiration and three of them improved after treatment. Five of the seven subjects demonstrated inefficient swallowing of thin liquids in at least one VFSS during the study period and six of the seven subjects had inefficient swallowing with at least 1 consistency during the study. All subjects demonstrated either unsafe or inefficient swallowing, or both, at least once during the study period.
Assessing prevalence and characteristics of oro‑bulbar involvement in children and adults with sma type 2 and 3 using a multimodal approach Trucco F. et al. [42]	Inclusion criteria: Genetically and clinically confirmed diagnosis of SMA.	67 Patients undergoing Nusinersen treatment 45 children 22 adult 11 untreated patients	*-Active Maximum mouth opening (AMMo);* *- Test of mastication and swallowing solid in children and adults (ToMASS);* *- Iowa oral Performance Instrument (IoPI);* *-The SMA health index;*	In SMA types 2 and 3, mouth opening and tongue strength were most affected, especially in children and sitters compared to walkers. Swallowing remained stable in nusinersen patients. In ambulant patients, chewing was comparable to that of healthy children and better than in sitters. Limited mouth opening and tongue strength correlated well with shorter eating times and reduced swallowing cycles. Three test sets further confirmed the interplay between tongue strength, mouth opening, and bolus processing in the oral phase of swallowing.
onasemnogene abeparvovec preserves bulbar function in infants with presymptomatic spinal muscular atrophy: a post-hoc analysis of the SPR1NT trial Shell R. D. et al. [34]	Inclusion criteria: Genetically and clinically confirmed diagnosis of SMA patients asymptomatic at the time of onasemnogene administration and < 6 postnatal weeks.	29 SMA patients 14 = 2 copies SMN2 followed until 18 months of age 15 = 3 copies SMN2 followed till 24 m. old	- Swallowing was evaluated at screening, with formal tests every 6 months starting at Month 6 and at study end (18 months for the two-copy cohort, 24 months for the three-copy cohort). Assessments were done via clinical bedside or fluoroscopic exams, based on standard practice and clinician discretion at each site; - *VFSS*	At baseline, no child exhibited clinical signs strongly suggestive of SMA (e.g., tongue fasciculation, hypotonia, areflexia). No physiologic swallowing impairment was identified, all children received full oral nutrition, and none had a history of pulmonary instability. At the final assessment, 100% (29/29) of children showed normal swallowing, received full oral nutrition, and did not require non-oral support. All 29 children met the composite endpoint of normal swallowing, full oral nutrition, and pulmonary stability.
Patients with spinal muscular atrophy type 1 achieve and maintain bulbar function following onasemnogene abeparvovec treatment McGrattan K. E. et al. [48]	Inclusion criteria: Genetically and clinically confirmed diagnosis of SMA type 1 patients received the therapeutic dose or equivalent of onasemnogene and where younger of 6 years old at the time of administration of treatment.	65 patients SMA type 1	- Data from three clinical studies were pooled from: START, STR1VEUS, and STR1VE-EU; - By clinical bedside examination; - *Fluoroscopic examination;* - Need for nutritional support was assessed via parent report or provider observation. Swallowing assessment: - at screening - every 6 months, starting at month 6 - At study end, at 18-mos or 24-mos post-infusion.	No issue in Swallowing function Baseline 88% [57] Endpoint 92% [59] 4 START 11 START 22 STR1VEUS 20 STR1VEUS 31 STR1VE-EU 29 STR1VE-EU Full oral nutrition: Baseline 85% [55] Endpoint 75% [49] 7 START 6 START 22 STR1VEUS 19 STR1VEUS 26 STR1VE-EU 24 STR1VE-EU
Assessing bulbar function in spinal muscular atrophy using patient-reported outcomes Young S.D. et al. [44]	Inclusion criteria SMA Patients undergoing Nusinersen treatment Exclusion criteria: unstable medical conditions	47 patients Male, n (%) 23 (48.9) SMA Type, n (%) 1 3 (6.4) 2 33 (70.2) 3 11 (23.4)	*-Voice handicap index;* *- Eating Assessment tool;* *- EK2;* *- RULM;* *- CHoP-INTEND.*	85% reported perceived swallowing issues primarily in 3/10 EAT-10 questions: swallowing pills (68.1%); food sticks to my throat (66.0%); swallowing solids (61.7%). In EK2, 64% of participants reported ineffective coughing, 64% required head support while driving or sitting in a wheelchair, 56% modified their food to eat, 72% couldn’t finish a meal in the same time as others, and 64% experienced swallowing issues, including choking.
Dysphagia and lung disease in children with spinal muscular atrophy treated with disease-modifying agents Chacko A. et al. [45]	All children with treated SMA types 1–3 Inclusion criteria: Children aged 0–18 y with symptom based SMA diagnosis (no neonatal screening), homozygous SMN1 gene alteration, chest CT, and available DMAs.	36 Patient (9 SMA 1; 14 TYPE 2; 13 TYPE 3). DMAs included nusinersen alone or in a combination with onasemnogene abeparvovec for type 1, and nusinersen alone for type 2/3.	*- Video fluoroscopy (* type 1 alone *);* *- The Penetration- Aspiration Scale (P-A score);* *- The Pediatric Functional oral Intake Scale (PFoIS);* *- Chest CT.*	VFSS: were available for 8 of 9 participants with type 1 all children demonstrated abnormal swallow Physiology. Most common impairments observed were multiple swallows to clear oral cavity (73.4%), delayed swallow initiation (65.0%), and abnormal pharyngeal residue (50.0%).No pattern was observed between impaired swallow physiology and non-oral feeding status or P-FoIS. Five participants were non-oral, despite no observed penetration/aspiration.
Risdiplam improves subjective swallowing quality in nonambulatory adult patients with 5qspinal muscular atrophy despite advanced motor impairment Brakemeier S. et al. [38]	Inclusion criteria: Genetically and clinically confirmed diagnosis of SMA type 2 or 3; non-ambulatory adults without a PEG and no prior SMN- therapy.	25 adult patients SMA type 2 24 SMA type 3 1 11 M 14 F	-*Sydney Swallow Questionnaire (SSQ);* *- Amyotrophic Lateral Sclerosis Functional Rating Scale Revised (b-ALSFRSR);* *- Hammersmith Functional Motor Scale Expanded (HFMSE);* *- RULM*.	Subjective swallowing quality, as measured by the SSQ, improved in adult non-ambulatory patients with SMA types 2 and 3 after 12 months of treatment with risdiplam. The SSQ score improved signifcantly from T0 to T2, with 15 patients (83%) reporting improvement and 3 (17%) reporting a decline within this period. There was no signifcant change in SSQ scores from T0 to T1.

**Table 2:** General comparison between the two study groups

Untreated study group [9]	Treated study group [14]
SMA Type – Number of studies	SMA Type & Therapies – Number of studies
◾ SMA I = 1 ◾ SMA II = 3 ◾ SMA II/III = 3 ◾ SMA I/II/III/IV = 2	◾ SMA I = 6 ◾ SMA II = 1 NUSINERSEN = 6 ◾ SMA II/III = 3 RISDIPLAM = 2 ◾ SMA I/II/III = 2 oNASEMNoGENE = 5 ◾ *N*° of SMN2 CoPIES = 2 NUSINERSEN and RISDIPLAM = 1

Used outcome Measure [number of studies]	Used outcome Measure [number of studies]
• Questionnaire for swallowing difficulties [3] • Diagnostic list of dysphagia e dysarthria in NMD [3] • Video fluoroscopic [3] • The Functional oral Intake Scale (FoIS) [2] • Test of mastication and swallowing solid [2] • Muscle Ultrasound [2] • Dysphagia limit [1] • Active maximal mouth opening [1] • Questionnaire of feeding difficulties [1] • Electromyography (EMG) [1] • Magnetic resonance imaging (MRI) bulbar muscle [1] • Neuromuscular disease swallowing status scale (NdSSS) [1] • 6 min Mastication Test (6MMT) [1] • Self-compiled questionnaire on swallowing for different type of food [1] • Timed test swallowing [1]	• Video fluoroscopic VFSS [5] + Fluoroscopic [2] • The Paediatric Functional oral Intake Scale (P-Fois) [3] • oral and Swallowing Abilities Tool (orSAT) [2] • Penetration aspiration scale (PAS) [3] • *Egen Klassifikation Scale version 2* (EK2) [2] • Eating assessment tool-10 [1] • Active maximum mouth opening [1] • Test of mastication and swallowing solid in children and adult [1] • Iowa oral performance instrument (IoPI) [1] • The SMA health index-it [1] • The Laryngoscopic Evaluation of Swallowing (FEES) [1] • Neuromuscular disease swallowing status scale (NdSSS) [1] • Sydney Swallow Questionnaire (SSQ) [2] • Amyotrophic Lateral Sclerosis Functional Rating Scale Revised (b-ALSFRSR) [2]

Legend: SMA: Spinal Muscular Atrophy; NMD: Neuromuscular Disease

The studies included patients with a variety of SMA types. Seven studies focused solely on SMA type I [7, 29–34], while four studies included patients with only type II [21, 35–37]. Additionally, six studies investigated patients with types II and III [38–43], two with type I, II and III [44, 45], and two studies encompassed all four SMA types (I, II, III, and IV) [46, 47]. Notably, two studies did not specify the SMA type but reported the number of SMN2 gene copies [48, 49]. Nusinersen was the most common treatment in the treated groups (*n* = 6) [6, 29, 30, 37, 40, 42], followed by onasemnogene (*n* = 4) [30, 33, 34, 48] and Risdiplam (*n* = 2) [37, 38]. Interestingly, one study used both Nusinersen and Risdiplam [49], while another used both Nusinersen and onasemnogene [45].

In both the group of articles concerning untreated patients and the group comprising treated patients, video fluoroscopic or fluoroscopic swallow study (VFSS) [7, 29–31, 34, 36, 41, 45, 48, 49] was utilized as a measure of outcome, even in a different proportion, in addition to various questionnaires administered to patients or their caregivers. VFSS, once a relatively uncommon outcome measure, became the predominant choice following the introduction of GBTs, marking a significant evolution from past clinical practices and research.

**Fig. 1:**
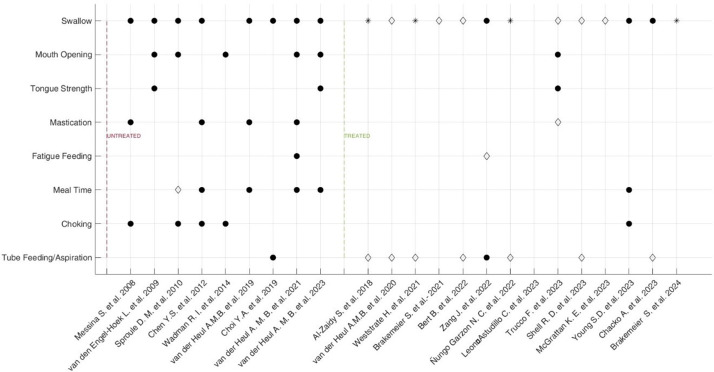
Distribution and severity rate changes of swallowing and swallowing related impairments before and after gene-based therapies introduction. Legend: ● Loss of function; ◊ Stability; ⁕ Gain of function

Figure 1 depicts the distribution of swallowing and swallowing-related impairments across the included studies, highlighting changes observed before and after gene therapy introduction.

## Discussion

In light of recent breakthroughs in SMA therapeutics, with the advent of effective treatments and a burgeoning pipeline of future advancements, ensuring the continuous acquisition and swift dissemination of clinical data, coupled with the corresponding adaptation of treatment protocols, is paramount for optimizing patient outcomes [50, 51]. Despite the promise of GBTs for improving various functions in SMA, a critical knowledge gap regarding swallowing function remains. This lack of robust research hinders the development of evidence-based protocols for managing swallowing difficulties in this patient population [52].

Swallowing dysfunction is a characteristic impairment in SMA, particularly affecting individuals with type 1.

In these patients, dysphagia can be caused by the weakness of the muscles involved in the swallowing mechanism, leading to alterations in the oral and pharyngeal phases of swallowing. This condition may also arise due to craniofacial alterations and weakness of the head and neck muscles, which compromise control of the head and trunk and hinder the adoption of compensatory protective postures [46]. Additionally, fatigue during feeding, manifested by longer meal times and consequently reduced food intake, deficits in mouth opening, and contractures of the masticatory muscles contribute to dysphagia [43]. The weakness of airway protection reflexes and gastroesophageal reflux (GER) also play significant roles [35].

The most common signs and symptoms of dysphagia in SMA include cough, difficulty managing secretions, gurgling voice, reduction of oral movements, primitive oral reflexes, insufficient oral hygiene, delayed initiation of the swallowing act, uncoordinated chewing and swallowing, fatigue, oral and nasal regurgitation, choking, low-grade fever, and changes in vital signs [31, 47]. The consequences and impact of dysphagia on the physical, emotional, and social dimensions of patients with SMA are profound. Common consequences include loss of appetite, weight loss, malnutrition, isolation, and depression, as mealtime plays a crucial role in social interactions [43].

From a clinical risk perspective, dysphagia can lead to penetration events, where the bolus enters the laryngeal vestibule above the vocal cords and may be expelled via defense mechanisms. More severe is laryngeal aspiration, where the bolus passes into the lower airways below the vocal cords and might be coughed out. A direct consequence of these events is the potential onset of aspiration pneumonia, caused by pharyngeal incoordination, prolonged latency of the swallowing reflex, reduced laryngeal sensitivity, and diminished airway defense reflexes [31].

Given the inherently degenerative nature of SMA, which often necessitated non-oral nutrition and resulted in an absence of swallowing skills in patients, it was deemed valuable to focus on pre- and post-pharmacological therapy assessments. The phenotypic shift in patients is readily apparent in the findings of our scoping review. Several factors indicate a change in the needs and abilities that these patients achieve throughout the different stages of their lives. The most striking difference is the marked disparity in the number of studies investigating swallowing abilities in SMA type 1 patients in the pre- and post-treatment era.

Indeed, prior to the advent of these therapies, research had primarily focused on SMA types 2 and 3 [21, 35, 36, 40, 41, 43, 46, 47], as evaluating swallowing aspects in type 1 [29] patients was challenging, if not impossible, in many cases, because compromised by the necessary implantation of a tracheostomy [53].

Studies conducted before the advent of GBTs painted a concerning picture of swallowing function in SMA patients. The functions involved in the preparatory oral phase (fatigue, mastication, tongue strength, mouth opening), swallowing itself, and meal clearance time were frequently reported as significantly compromised across all SMA types. However, due to the severe bulbar dysfunction, particularly in SMA 1, evaluation of these functions was often impossible, leading to a scarcity of data on aspiration risk.

Swallowing dysfunction was a prominent feature in the pre-GBTs group. Patients most commonly reported difficulties with chewing (mastication), choking on solids and liquids, and abnormal tongue movements during eating. Swallowing itself was weak, and patients required repeated attempts to clear their mouths. Limited hyoid and mandibular movement further contributed to these problems, often manifesting as difficulty opening the mouth. Interestingly, Choi et al. [29] was the only study in this group to report pharyngeal phase difficulties in SMA patients. This finding may be related to their focus on SMA type 1, which is known to have a more severe phenotype [54]. In their study, patients with SMA type 1 typically required tube feeding by a median age of six months due to significant swallowing dysfunction that often worsened before 12 months.

Aspiration problems were more frequently reported in SMA patients from the second group of studies, possibly due to the focus on improvement or stabilization in these studies.

Among the studies in this second group, only Zang et al. [33] reported worsening with aspiration phenomena and non-oral nutrition. In contrast, all other studies included reported a trend of stabilization [7, 30–32, 45, 48] or improvement, even in situations where respiratory support and percutaneous endoscopic gastrostomy (PEG) were recommended at baseline pre-treatment [37, 49].

one could speculate that the decline in yearly tracheotomy rates in recent years, along with changes in the characteristics of this patient group, may have influenced these results.

Both groups included a mix of adult and paediatric participants with varying disease severity. Further compounding this issue, a recurrent methodological critical gap emerged: the absence of a standardized, validated assessment tool for this population. As a result, the studies employed a wide range of instruments, making it difficult to compare and analyse the findings effectively.

In studies conducted prior to the advent of GBTs, outcome measures primarily relied on questionnaires completed by caregivers or assessment scales administered by professionals [21, 35, 41, 43, 46]. Endurance tests, such as the 6-Minute Mastication Test (6MMT) [40], masticatory muscle ultrasound [40, 41], or MRI [46] were less frequently employed. Video fluoroscopy (VFSS) was utilized in only 3 of these 9 studies [29, 36, 41]. This trend shifted in studies involving treated patients. VFSS became the dominant investigative method, employed in 7 out of the 14 studies [7, 30, 31, 34, 45, 48, 49]. This shift highlights a trend towards greater objectivity and reliability in the outcome measures employed.

Beyond the previously mentioned measures, in both study groups were used other assessment tools, including the P-fois/fois [7, 40, 41, 45, 49], the Test of Mastication and Swallowing Solid (TMSS) [40–42], Active Maximal Mouth opening (AMMo) [40, 42], and the Neuromuscular Disease Swallowing Status Scale (NdSSS) [29, 33].

In recent years, recognizing the persistent heterogeneity in outcome measures, researchers have developed additional tools for evaluating swallowing function in SMA. These include the oral Swallowing Assessment Tool (orSAT) [32, 33] and the SMA Health Index-IT (SMAHI-IT) [42].

The distribution of GBTs in the included studies mirrored the timeline of approval for different SMA gene therapies. Nusinersen (marketed as Spinraza) received approval in 2016, followed by onasemnogene abeparvovec (Zolgensma, formerly AXS-101) in 2019 and Risdiplam in August 2020 for adults and children ≥ 2 months old [26, 55, 56]. This suggests Nusinersen as the most investigated therapy to date due to its earlier availability.

In a study by S. Brakemeier et al. [38], Risdiplam treatment significantly improved swallowing quality in adult non-ambulatory patients with SMA types 2 and 3 after 12 months. Conversely, the same research group, using the same evaluation method, found that Nusinersen treatment in a prior study did not improve swallowing, but rather preserved bulbar function [39]. However, current evidence from limited data does not allow to definitively determining the most effective GBT for improving swallowing function. Although research indicates that Nusinersen alleviates bulbar symptoms in SMA patients [57–59], the specific evidence regarding its effect on swallowing function remains less definitive and comprehensive. Direct comparisons of these therapies’ impact on swallowing function are challenging but crucial for future studies to optimize clinical practice. This will allow determining which therapy offers the greatest benefit for swallowing, ultimately supporting more individualized treatment decisions.

## Conclusion

Swallowing dysfunction is a significant challenge in SMA patients, particularly those with most severe forms. However, the recent advent of effective GBTs has introduced a new era of management for this disease. This scoping review highlights a critical knowledge gap regarding the impact of these therapies on swallowing function.

Studies conducted prior to GBTs painted a concerning picture, with compromised swallowing function across all SMA types. However, methodological limitations, and a lack of standardized assessments made comparisons with the post-treatment era difficult.

The post-treatment era demonstrated a potential shift in swallowing function. Studies reported trends towards stabilization or improvement, even in previously tube-fed patients. This finding may be related to a decrease in annual tracheotomy rates and the phenotypic evolution of the treated population.

The limited data currently available makes it challenging to definitively determine the most effective GBT for swallowing function. Future research should prioritize the identification of the most beneficial treatment for individual patients on swallowing function. This, together with the development and implementation of specifically validated swallowing assessments will optimize clinical practice and patient outcomes in SMA.

## Data Availability

Data will be available on request to the corresponding author.
